# Global trends in gastric and colorectal cancer, 1990 to 2021: A population-based observational study using GBD 2021 data

**DOI:** 10.1097/MD.0000000000048276

**Published:** 2026-04-10

**Authors:** Ling Yu, Wei Xu

**Affiliations:** a Department of General Practice, The Affiliated Yongchuan Hospital of Chongqing Medical University, Chongqing, China; b Department of Gastrointestinal Surgery, The Affiliated Yongchuan Hospital of Chongqing Medical University, Chongqing, China.

**Keywords:** colorectal cancer, gastric cancer, Global Burden of Disease

## Abstract

Gastric and colorectal cancers are among the most prevalent gastrointestinal malignancies worldwide and represent significant global public health concerns. Their epidemiological profiles vary substantially across regions and sociodemographic contexts. Despite advances in screening and treatment, both types of cancer continue to cause considerable morbidity and mortality. A comprehensive understanding of these temporal trends and future trajectories is critical for developing effective cancer control policies. Data from the Global Burden of Disease Study 2021 were used to systematically evaluate age-standardized incidence rates (ASIRs), prevalence rates, mortality rates (ASMRs), and disability-adjusted life-year rates for gastric and colorectal cancers between 1990 and 2021. Temporal trends were assessed using joinpoint regression and ARIMA models. Decomposition analysis was performed to differentiate among the effects of population growth, aging, and epidemiological changes. Finally, trend-based projections of the global burden through 2050 were performed. Between 1990 and 2021, both ASIR and ASMR for gastric cancer declined globally, particularly in high Socio-demographic Index countries. Conversely, colorectal cancer demonstrated an increasing trend in ASIR and age-standardized prevalence rates, although ASMR and disability-adjusted life-year rates decreased. Countries with a high Socio-demographic Index exhibited a higher incidence of colorectal cancer but benefited from lower mortality, likely reflecting differences in healthcare capacity. Males experienced a consistently higher burden of both cancers. Trend-based projections suggest a continued decline in the burden of gastric cancer, whereas the incidence of colorectal cancer may remain relatively stable or increase modestly, with mortality continuing to decline. Despite declining mortality rates, gastric and colorectal cancers remain a substantial global health burden, with distinct epidemiological patterns. Enhancing early detection strategies, such as gastroscopy and colonoscopy, along with dietary modifications and *Helicobacter pylori* eradication in high-risk populations, may help mitigate the future disease burden and inform health policy interventions.

## 1. Introduction

Cancer is a major public health challenge worldwide and is the third leading cause of death globally, accounting for an estimated 1 in 7 deaths in 2021.^[1,2]^ Among these, digestive tract tumors are relatively prevalent, with gastric and colorectal cancers constituting the most common digestive tract malignant tumors worldwide. Their epidemiological characteristics are significantly heterogeneous, owing to differences in geographic regions, socioeconomic levels, and lifestyles. In 2020, global cancer statistics delineated that gastric cancer accounted for approximately 1.09 million new cases and 769,000 deaths, ranking fourth among cancer-related deaths. Colorectal cancer, on the other hand, is the second leading cause of cancer-related deaths, with 1.93 million new cases and 935,000 deaths, collectively accounting for 26.3% of global cancer-related deaths.^[3]^ Notably, the incidence of gastric cancer is largely concentrated in East Asia, accounting for 58% of global cases, Eastern Europe, and the Andean region.^[4]^ Meanwhile, the burden of colorectal cancer is particularly high in industrialized countries, with incidence rates in Australia and Western Europe being 8 to 10 times higher than in West Africa.^[5]^

Gastric cancer pathogenesis is closely related to *Helicobacter pylori* infection (contribution of approximately 89%), high salt intake, and smoking.^[6]^ The global age-standardized incidence rate (ASIR) has been decreasing at an average annual rate of 1.3% over the last 3 decades owing to the widespread adoption of refrigerators, improvements in dietary structure, and the implementation of *H pylori* eradication strategies.^[7]^ However, the age-standardized mortality rate (ASMR) of gastric cancer exhibits significant geographic variation. For instance, Korea has improved its 5-year survival rate to 75% through a national screening program,^[8]^ whereas ASMR continues to rise in sub-Saharan Africa due to delayed diagnosis.^[9]^ The global ASIR for colorectal cancer increased by 9.5% between 1990 and 2019.^[10]^ The atypical annual increase of 2% in early-onset colorectal cancer in individuals under 50 years of age has garnered extensive academic attention,^[11]^ which may be associated with the increased consumption of processed foods, intestinal flora dysbiosis, and exposure to environmental endocrine disruptors.^[12]^ Geographically, the ASIR of gastric cancer in Korea (39.6/100,000) is 10 times higher than that in Niger (3.2/100,000),^[13]^ whereas the incidence of colorectal cancer in Hungary (45.3/100,000) is 13 times higher than that in Guinea (3.3/100,000).^[14]^

Despite advances in preventive and control strategies, such as endoscopic screening and X-ray screening in Japan, which reduces gastric cancer mortality by 67%, as well as colonoscopy screening, which reduces the risk of colorectal cancer by 18%,^[15]^ gastric and colorectal cancers, as prevalent gastrointestinal tract tumors, continue to add to the global burden of disease.

Therefore, this study aimed to systematically assess the global burden of gastric and colorectal cancers by analyzing ASIRs, prevalence rates (ASPRs), ASMRs, and disability-adjusted life-year rates (ASDRs) across regions, age groups, and sexes using data from the Global Burden of Disease (GBD) 2021 study. In addition, long-term temporal trends from 1990 to 2021 were examined, and future burden patterns were explored using time-series forecasting methods.

Compared with previously published GBD-based analyses, this study provides updated estimates based on the most recent GBD 2021 data, offers a comprehensive side-by-side comparison of gastric and colorectal cancers, and integrates trend analysis with forecasting to highlight potential future trajectories at the global, regional, and national levels.

## 2. Methods

This study systematically analyzed the epidemiological characteristics of gastric and colorectal cancers based on data derived from the GBD Study 2021 database. The GBD database, which is a collaboration between the World Health Organization and several global research institutes, provides multidimensional health data on disease burden, mortality rate, and incidence rate worldwide across countries and regions from 1990 to 2021. Using this database, the ASIR, ASPR, ASMR, and ASDR for gastric and colorectal cancers were extracted to explore epidemiological trends, regional variations, and changes in these 2 cancers over time. This study was based on publicly available, deidentified data from the GBD 2021 database; therefore, ethical approval and informed consent were waived.

### 2.1. Data sources and screening

This study used the publicly available GBD version 2021 dataset, which covers epidemiological data on gastric and colorectal cancers worldwide. The following key metrics were extracted:

ASIR: The number of new cases of gastric or colorectal cancer per 100,000 population per year, age-standardized to eliminate the effect of age differences.
ASPR: The prevalence of diagnosed gastric or colorectal cancer per 100,000 population per year, age-standardized to minimize the effect of age differences.
ASMR: The number of deaths attributable to gastric or colorectal cancer per 100,000 population per year, age-standardized to assess differences between regions.
ASDR: The number of disability-adjusted life years (DALYs) per 100,000 population per year due to a particular type of gastric or colorectal cancer, age-standardized to facilitate cross-national and cross-regional comparisons.

Global, continental, national, and regional data on gastric and colorectal cancer indicators for the period 1990 to 2021 were sourced from the GBD online query platform, and selected data were stratified by gender and age group to allow for an in-depth analysis of differences between different groups.

### 2.2. Data processing and statistical analysis

The data retrieved from the GBD database were standardized and had a high degree of accuracy and comparability. The primary analyses performed on the raw data included descriptive statistics, trend analysis, and regional comparisons. The detailed steps of the statistical analysis are as follows:

#### 2.2.1. Regional variation analysis

To elucidate differences in the geographical distribution of gastric and colorectal cancers, comparative analyses were conducted for ASIR, ASPR, ASMR, and ASDR across regions (e.g., continents), countries, and regions with different Socio-demographic Index (SDI) levels. Furthermore, the burden of gastric and colorectal cancer in different regions and countries was evaluated.

#### 2.2.2. Gender and age-specific analysis

To accurately assess the impact of sex and age on gastric and colorectal cancer, indicators were separately calculated for males and females, as well as across age groups. Comparing ASIR, ASPR, ASMR, and ASDR enabled the evaluation of differences in disease burden between gastric and colorectal cancers across sex and age groups and the susceptibility and mortality risks associated with these 2 cancers in different populations.

#### 2.2.3. Temporal trend analysis

Initially, annual time-series graphs for gastric and colorectal cancers were plotted using ASIR, ASPR, ASMR, and ASDR. These plots allowed the visualization of changes in the incidence and mortality of these cancers globally and in specific regions (e.g., high-income countries, low-income countries). Linear regression and ARIMA models were fitted to each dataset to assess the average annual growth rate and trends in the incidence and mortality of gastric and colorectal cancers.

#### 2.2.4. Data analysis tools and methods

The statistical software **R** (R Foundation for Statistical Computing, Vienna, Austria), **Joinpoint Regression Program** (version **6.0.1**; National Cancer Institute, Bethesda), and **Microsoft Excel** (Microsoft Corporation, Redmond) were utilized in this study for data organization, visualization, and analysis. Briefly, the data extracted from the GBD database were preprocessed to ensure consistency and completeness. Descriptive statistics were then applied to summarize and present indicators and to visualize differences in the burden of disease across different regions, sexes, and age groups. In addition, statistical methods, such as linear regression analysis and time-series analysis, were used to assess the prevalence of gastric and colorectal cancers at different time points and in different groups. Joinpoint regression analysis was conducted using the **Joinpoint Regression Program** (version **6.0.1**; National Cancer Institute, Bethesda).

The maximum number of joinpoints was set to 4, and model selection was based on the Monte Carlo permutation test with a significance level of .05.

Annual percent change and average annual percent change were calculated with corresponding 95% confidence intervals. Time-series forecasting was performed using autoregressive integrated moving average (ARIMA) models implemented in R (forecast package, CRAN, R Foundation for Statistical Computing, Vienna, Austria).

Stationarity of each time series was assessed using the augmented Dickey–Fuller test, and first-order differencing was applied when necessary.

Model parameters were selected using the auto.arima() function based on the Akaike Information Criterion corrected for small sample size.

Model adequacy was evaluated through residual diagnostics, including the Ljung–Box test.

#### 2.2.5. Interpretation of results

This study aimed to elucidate the trends and differences in the incidence, mortality, and disease burden of gastric and colorectal cancers globally by analyzing epidemiological data. By analyzing the ASIR, ASPR, ASMR, and ASDR, this study provides valuable insights for guiding global cancer prevention and control policies, especially in terms of differences between different levels of economic development and geographical regions. Significant gaps in preventive measures and early screening programs for gastric and colorectal cancers have been identified in certain regions. Future public health strategies should focus on high-risk regions and groups.

## 3. Results

### 3.1. Description of the gastric cancer and colorectal cancer burden

Globally, the burden of gastric cancer declined substantially between 1990 and 2021 (Table 1). Both the ASIR and ASMR showed sustained decreases over the study period, with a more pronounced decline observed in females than in males.

**Table 1:** Incidence, mortality, and average annual percentage change (AAPC) of gastric cancer and colorectal cancers by sex from 1990 to 2021.

	Incidence						Mortality					
	Case (n), 1990	Incidence (per 100,000), 1990	Case (n), 2021	Incidence (per 100,000), 2021	AAPC (1990–2021)	*P* value	Case (n)	Mortality (per 100,000), 1990	Case (n)	Mortality (per 100,000), 2021	AAPC (1990–2021)	*P* value
Gastric cancer												
Both	980,899.4 (891,306.8–1,072,236)	34.4 (30–38.7)	1,198,253.7 (1,025,012.9–1,363,511.2)	21.4 (17.5–25.2)	−1.763 (−1.889 to −1.638)	<.001	854,184.5 (772,885.1–939,972.7)	30.4 (26.4–34.5)	934,391.2 (803,724.6–1,052,110.3)	16.5 (13.4–19.4)	−2.184 (−2.304 to −2.064)	<.001
Male	628,157.7 (540,733.2 –705,759.3)	24.8 (22.6–27)	811,579.7 (661,665.7–960,683.9)	14.7 (12.5–16.7)	−1.596 (−1.743 to −1.448)	<.001	538,664.5 (459,835.1–612,037.6)	22 (20–24.2)	612,361.8 (496,042.3–724,331.1)	11.5 (9.9–13)	−2.057 (−2.194 to −1.919)	<.001
Female	352,741.7 (321,554.3–384,689.4)	16.6 (15.1–18.1)	386,674.1 (334,919.8–430,625.8)	8.8 (7.7–9.8)	−2.098 (−2.239 to −1.956)	<.001	315,520 (286,765.7–345,466.3)	15 (13.6–16.5)	322,029.4 (278,675.5–357,436.1)	7.3 (6.3–8.1)	−2.399 (−2.504 to −2.295)	<.001
Colorectal cancer												
Both	916,583.5 (866,238.3–951,895)	27.3 (25.9–28.5)	2,099,366.2 (1,935,826.8–2,235,151)	32 (29.3–34.8)	0.204 (0.158–0.25)	<.001	570,318.5 (536,544.8–597,668.7)	17.7 (16.7–18.7)	1,009,564 (931,471.5–1,071,258.1)	15.6 (14.3–16.8)	−0.716 (−0.804 to −0.628)	<.001
Male	469,762.6 (445,308.3–492,302.5)	24 (22.5–25)	1,206,473.7 (1,102,911.2–1,313,187.4)	25.7 (23.7–27.4)	−0.45 (−0.535 to −0.365)	<.001	287,710.7 (269,819.1–304,982.3)	15.6 (14.5–16.3)	561,531.3 (514,518.8–606,427.6)	12.6 (11.6–13.4)	−0.45 (−0.535 to −0.365)	<.001
Female	446,820.9 (414,136–472,552.6)	21.4 (19.7–22.6)	892,892.5 (792,207.5–962,761.2)	20.3 (18.1–21.9)	−0.186 (−0.253 to −0.119)	<.001	282,607.9 (258,886–301,416.1)	13.9 (12.7–14.8)	448,032.6 (395,786.6–483,743.3)	10.2 (9–11)	−1.057 (−1.137 to −0.977)	<.001

By contrast, the global ASIR of colorectal cancer increased modestly from 1990 to 2021 when both sexes were combined, whereas sex-stratified analyses indicated modest declines within each sex, highlighting differences between aggregated and stratified estimates (Table 1). Over the same period, colorectal cancer mortality declined consistently in both sexes, with a larger reduction observed among females than males.

**Figure 1.**
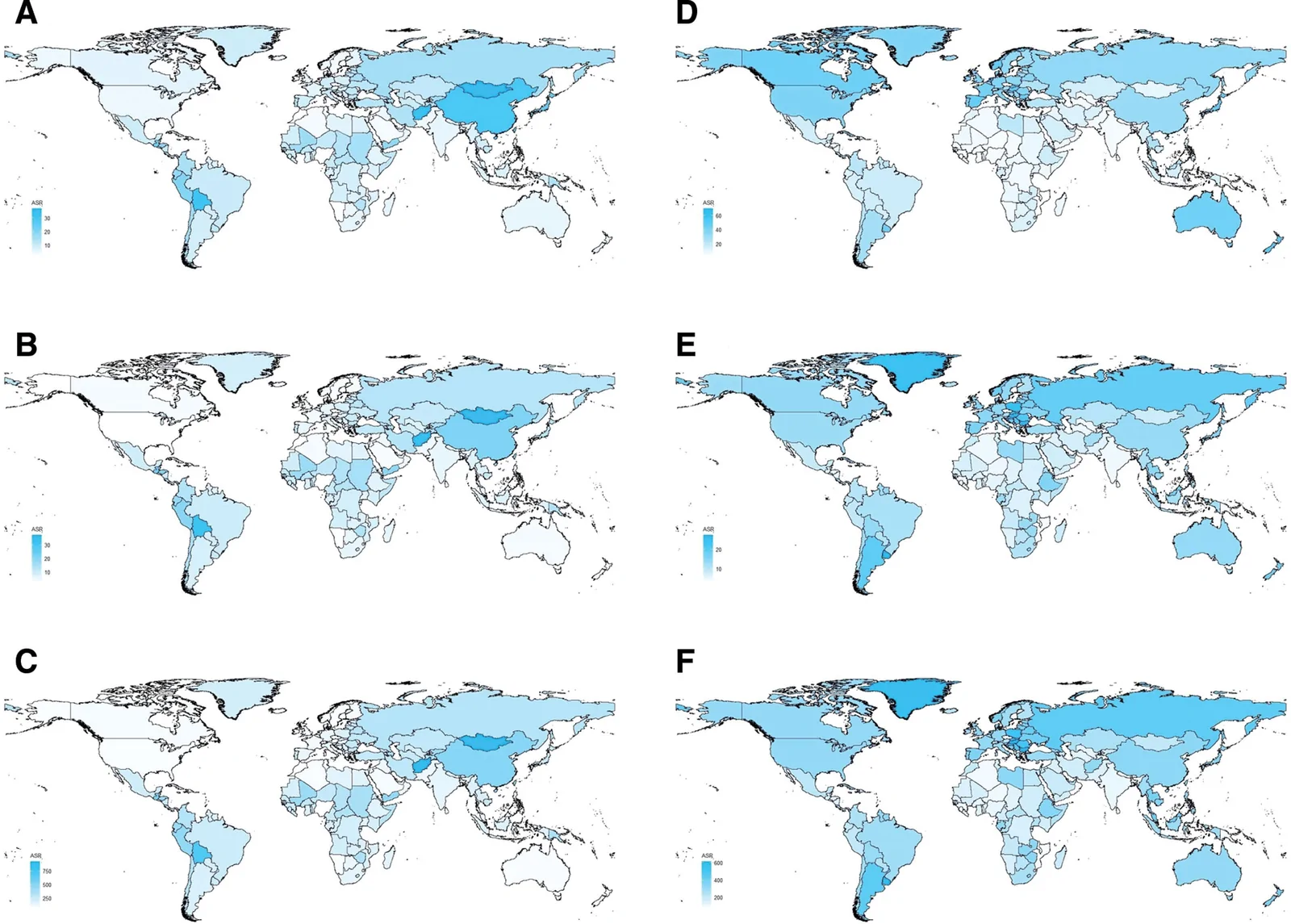
Global gastric cancer and colorectal cancer burden in 204 countries and regions in 2021. (A) ASIR of gastric cancer; (B) ASMR of gastric cancer; (C) ASDR of gastric cancer; (D) ASIR of colorectal cancer; (E) ASMR of colorectal cancer; (F) ASDR of colorectal cancer. ASDR = age-standardized disability-adjusted life-year rate, ASIR = age-standardized incidence rate, ASMR = age-standardized mortality rate.

Figure 1 illustrates marked geographic heterogeneity in the burden of gastric and colorectal cancers in 2021. Gastric cancer incidence, mortality, and DALY rates were highest in several East Asian countries, whereas colorectal cancer burden was concentrated in high-income regions of North America and Europe.

**Figure 2.**
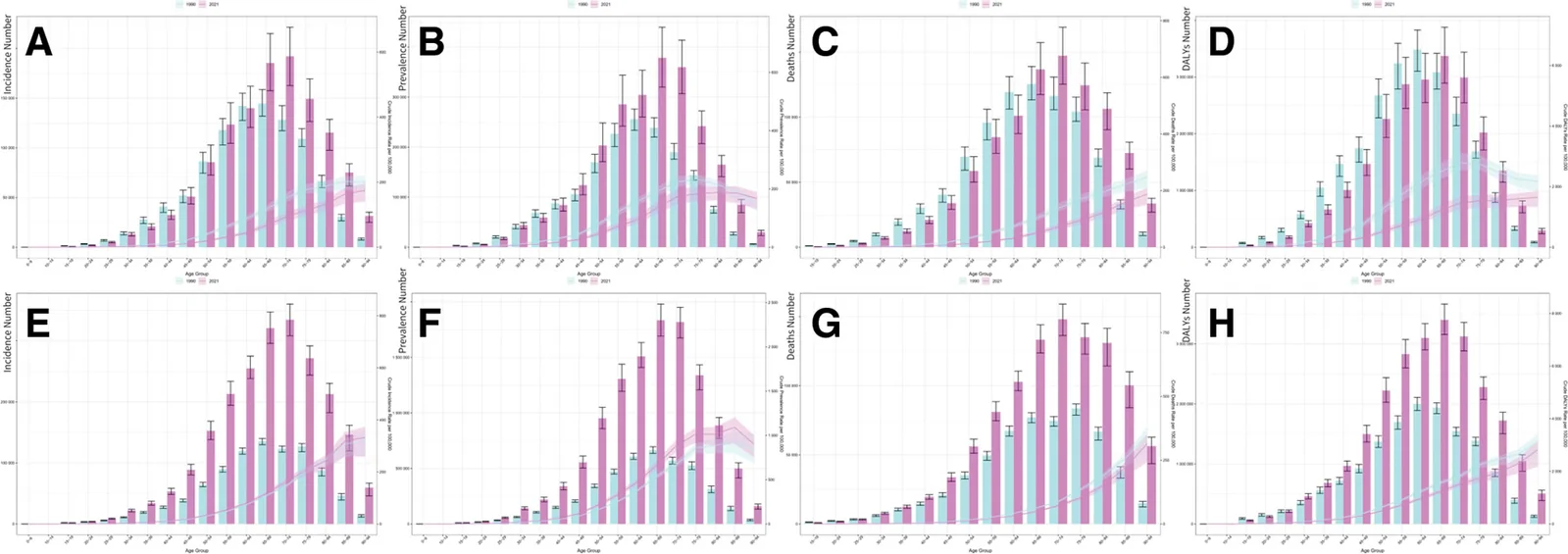
Comparison of incidence rates, prevalence rates, mortality rates, and DALY rates by age group and crude rates from 1990 to 2021. (A–D) Gastric cancer; (E–H) colorectal cancer. The black line and shaded area represent the 95% UI. DALYs = disability-adjusted life years, UI = uncertainty interval.

As shown in Figure 2, the incidence of both gastric and colorectal cancers increased with age. Compared with 1990, gastric cancer incidence and mortality declined across all age groups in 2021. By contrast, colorectal cancer incidence rates changed little across age groups; however, the absolute number of colorectal cancer cases and deaths increased substantially, largely driven by population aging.

**Figure 3.**
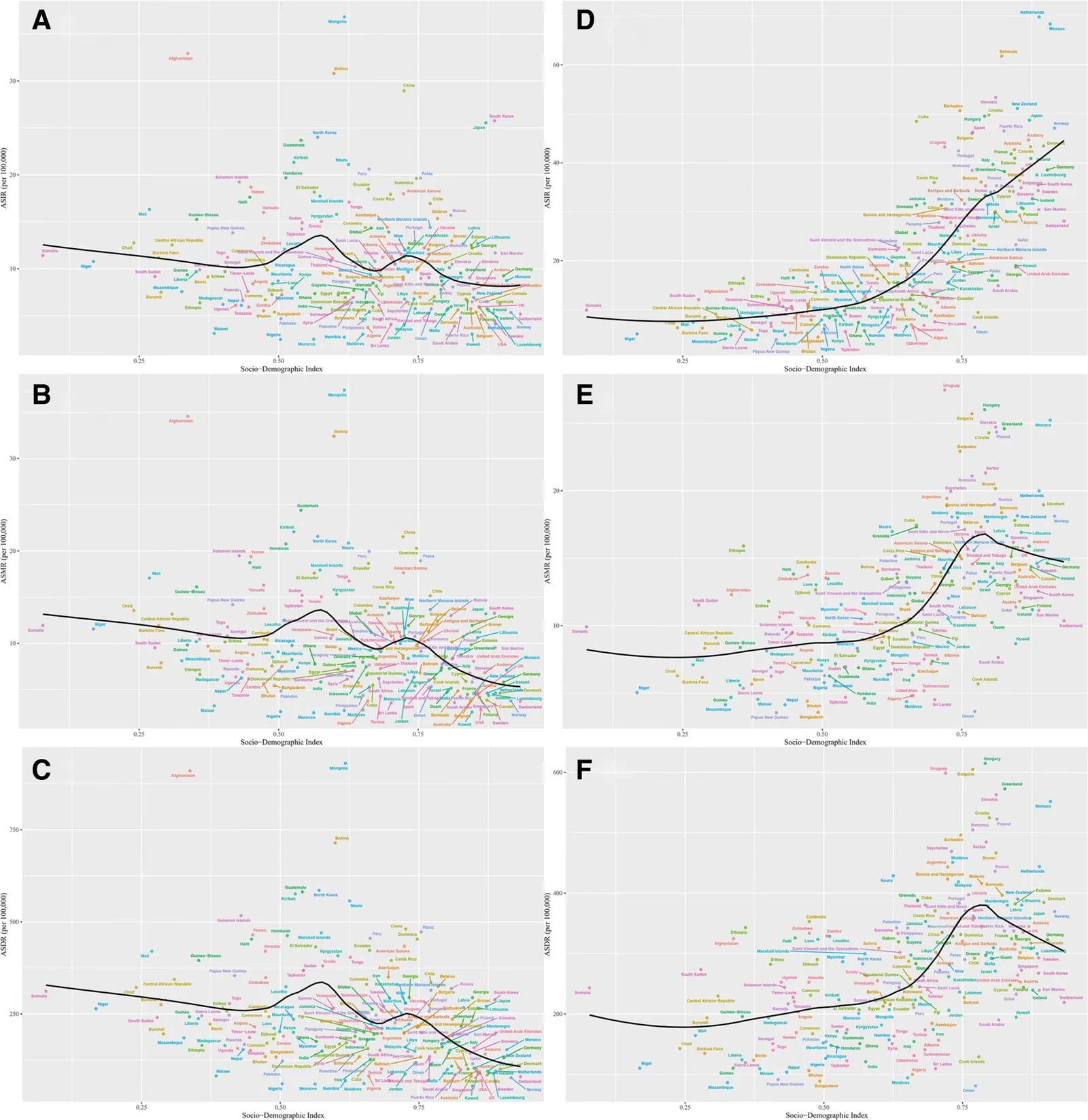
Age-standardized burden rate attributable to gastric cancer and colorectal cancer across 204 countries and regions by Socio-demographic Index, 2021. The black line represents a LOESS regression fitted to all data points. (A–C) Gastric cancer; (D–F) colorectal cancer. ASDR = age-standardized disability-adjusted life-year rate, ASIR = age-standardized incidence rate, ASMR = age-standardized mortality rate, LOESS = locally estimated scatterplot smoothing.

Figure 3 demonstrates distinct associations between the SDI and the burden of gastric and colorectal cancers. Gastric cancer incidence was generally lower in regions with higher SDI, whereas colorectal cancer incidence increased with SDI. Notably, in high-SDI regions, colorectal cancer mortality and DALY rates declined despite persistently high-incidence levels.

**Figure 4.**
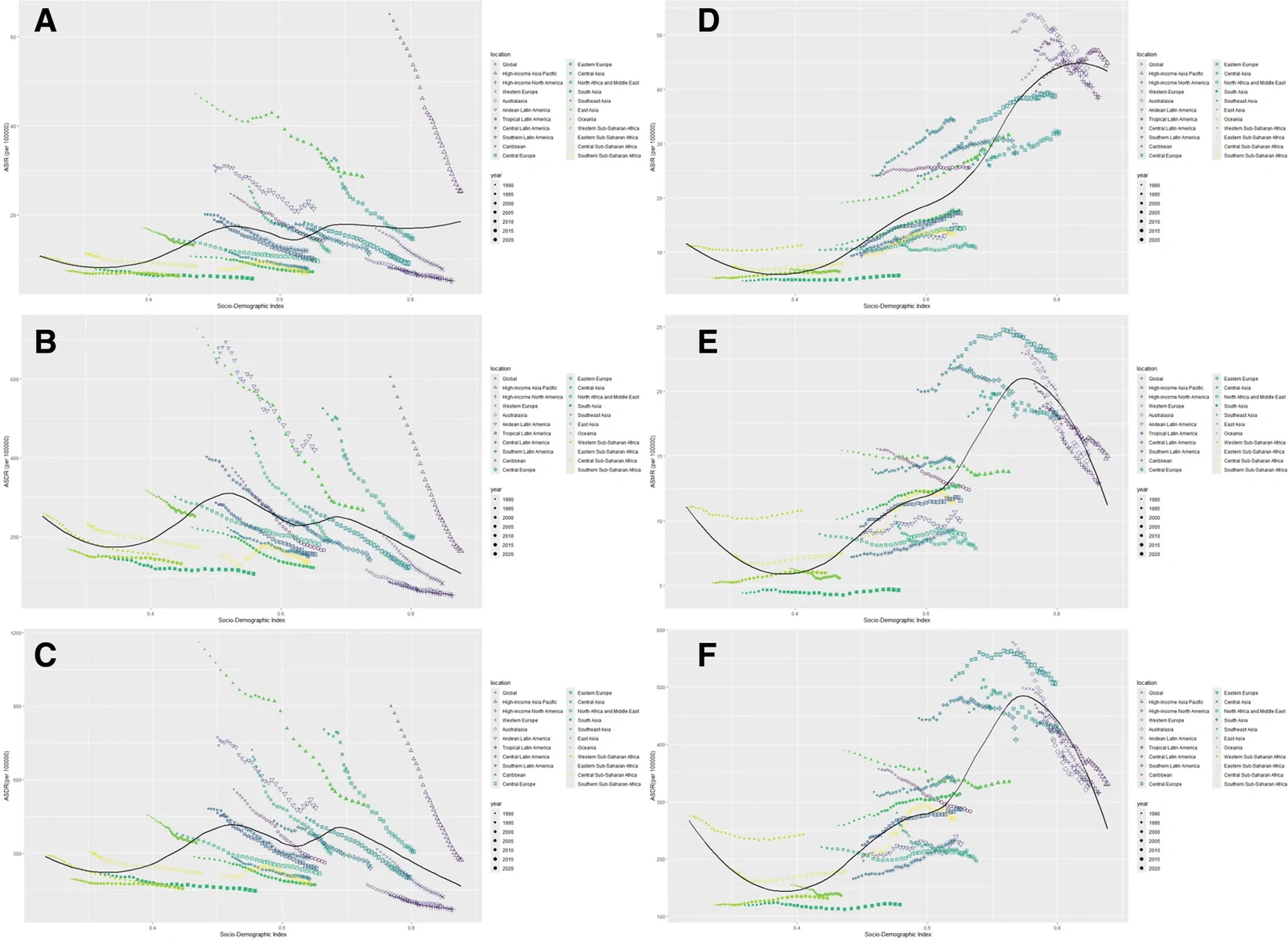
Age-standardized burden rates attributable to gastric cancer and colorectal cancer across 22 regions by Socio-demographic Index, 1990 to 2021. The black line denotes an adaptive LOESS regression fitted to all data points. (A–C) Gastric cancer; (D–F) colorectal cancer. ASDR = age-standardized disability-adjusted life-year rate, ASIR = age-standardized incidence rate, ASMR = age-standardized mortality rate.

Figure 4 shows that with increasing SDI, the incidence, mortality, and DALY rates of gastric cancer declined steadily, particularly in high-SDI regions. By contrast, colorectal cancer incidence increased with SDI, while mortality and DALY rates declined over time in high-SDI regions.

**Figure 5.**
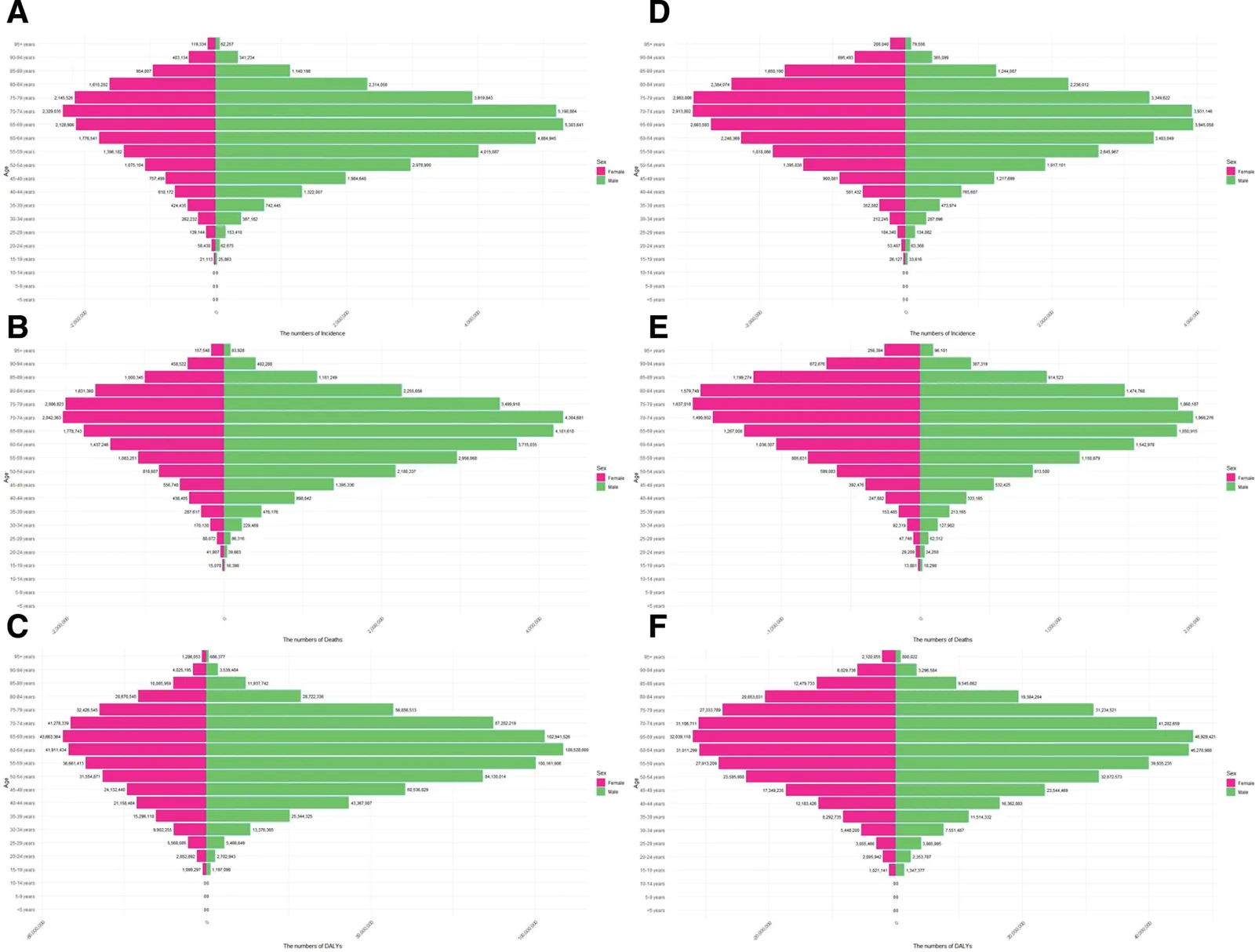
Comparison of disease burden between gastric cancer and colorectal cancer across different age groups in 2021. (A–C) Gastric cancer; (D–F) colorectal cancer. DALYs = disability-adjusted life years.

Figure 5 highlights substantial sex-specific differences in the burden of gastric and colorectal cancers. Incidence and DALY burdens were consistently higher in males than in females, particularly in older age groups, indicating a markedly higher disease burden among men for both cancer types.

#### 3.1.1. Decomposition analysis

**Figure 6.**
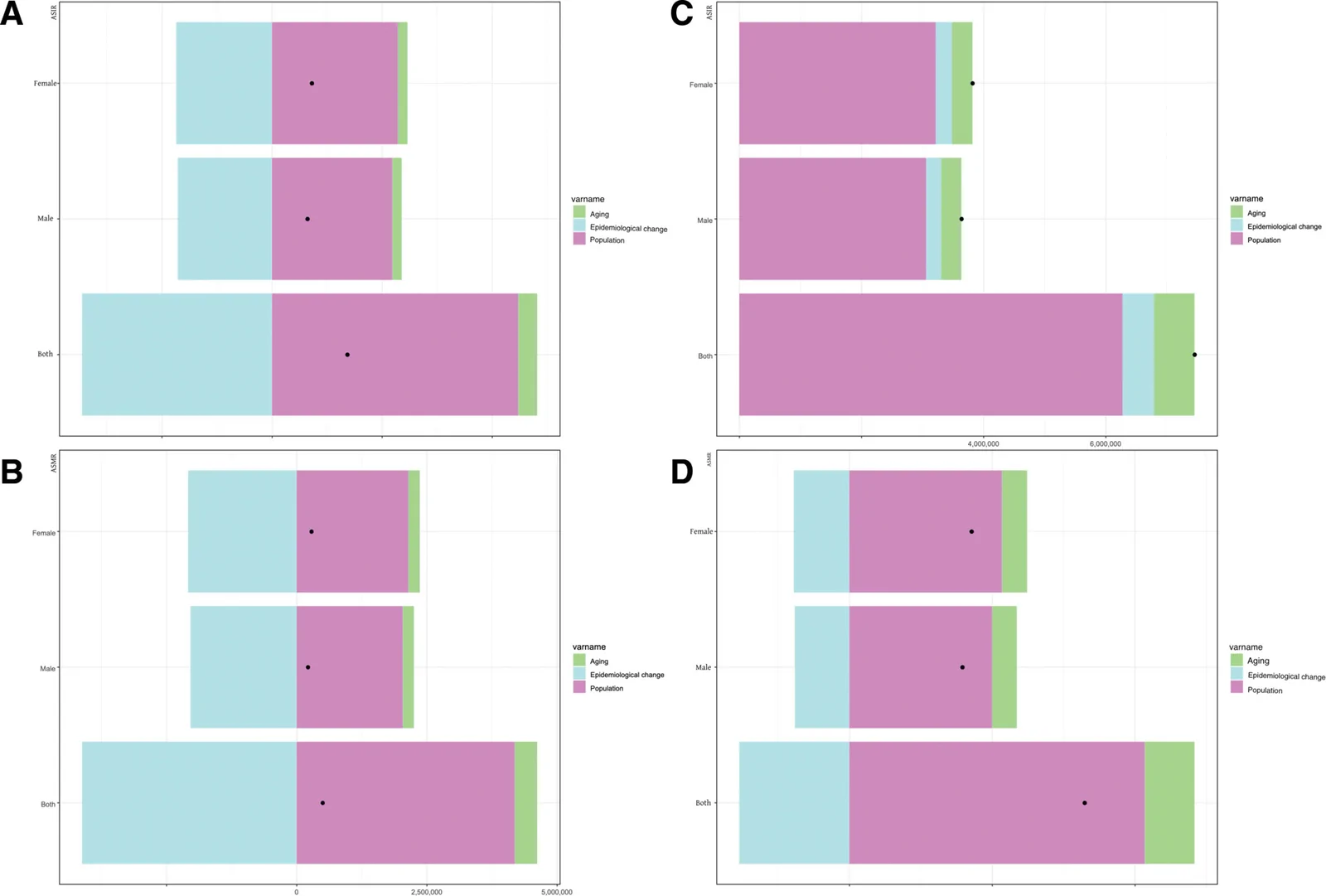
Decomposition analysis of gastric cancer and colorectal cancer in China and globally from 1990 to 2021. (A, B) Gastric cancer; (C, D) colorectal cancer. ASIR = age-standardized incidence rate, ASMR = age-standardized mortality rate.

Decomposition analysis indicated that changes in the burden of gastric cancer were primarily driven by epidemiological trends, whereas population growth was the dominant contributor to the increasing burden of colorectal cancer (Fig. 6).

#### 3.1.2. Joinpoint analysis of the gastric cancer and colorectal cancer burden

**Figure 7.**
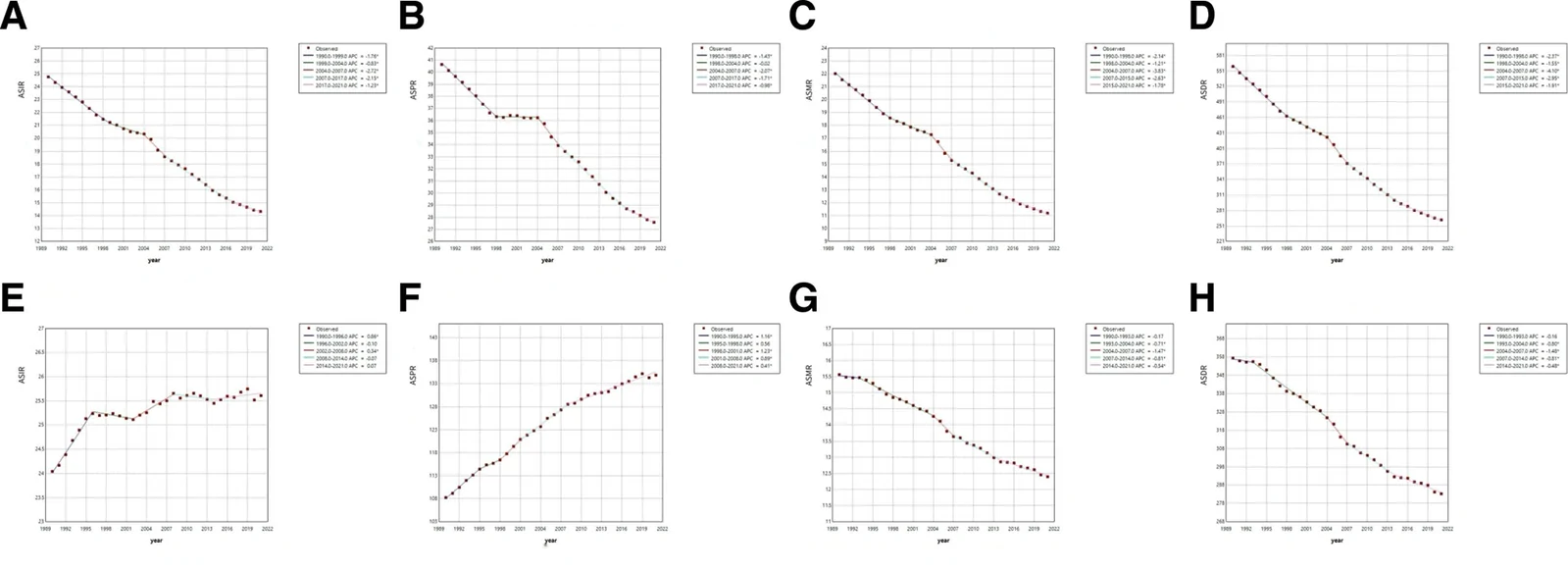
The APC of ASIR, ASPR, ASMR, and ASDR in gastric cancer (A–D) and colorectal cancer (E–H) from 1990 to 2021 (* indicates *P* value < .05, statistically significant). (A, E) ASIR; (B, F) ASPR; (C, G) ASMR; (D, H) ASDR. APC = annual percent change, ASDR = age-standardized disability-adjusted life-year rate, ASIR = age-standardized incidence rate, ASMR = age-standardized mortality rate, ASPR = age-standardized prevalence rate.

Joinpoint analysis revealed a sustained decline in the incidence, prevalence, mortality, and DALY rates of gastric cancer since 1990, with the most rapid decreases occurring in the mid-2000s (Fig. 7A–D). By contrast, colorectal cancer incidence showed an overall increasing trend, whereas mortality and DALY rates declined over time, particularly after the early 2000s (Fig. 7E–H).

### 3.2. Prediction of ASIR, ASPR, and ASMR for gastric and colorectal cancer through 2050

**Figure 8.**
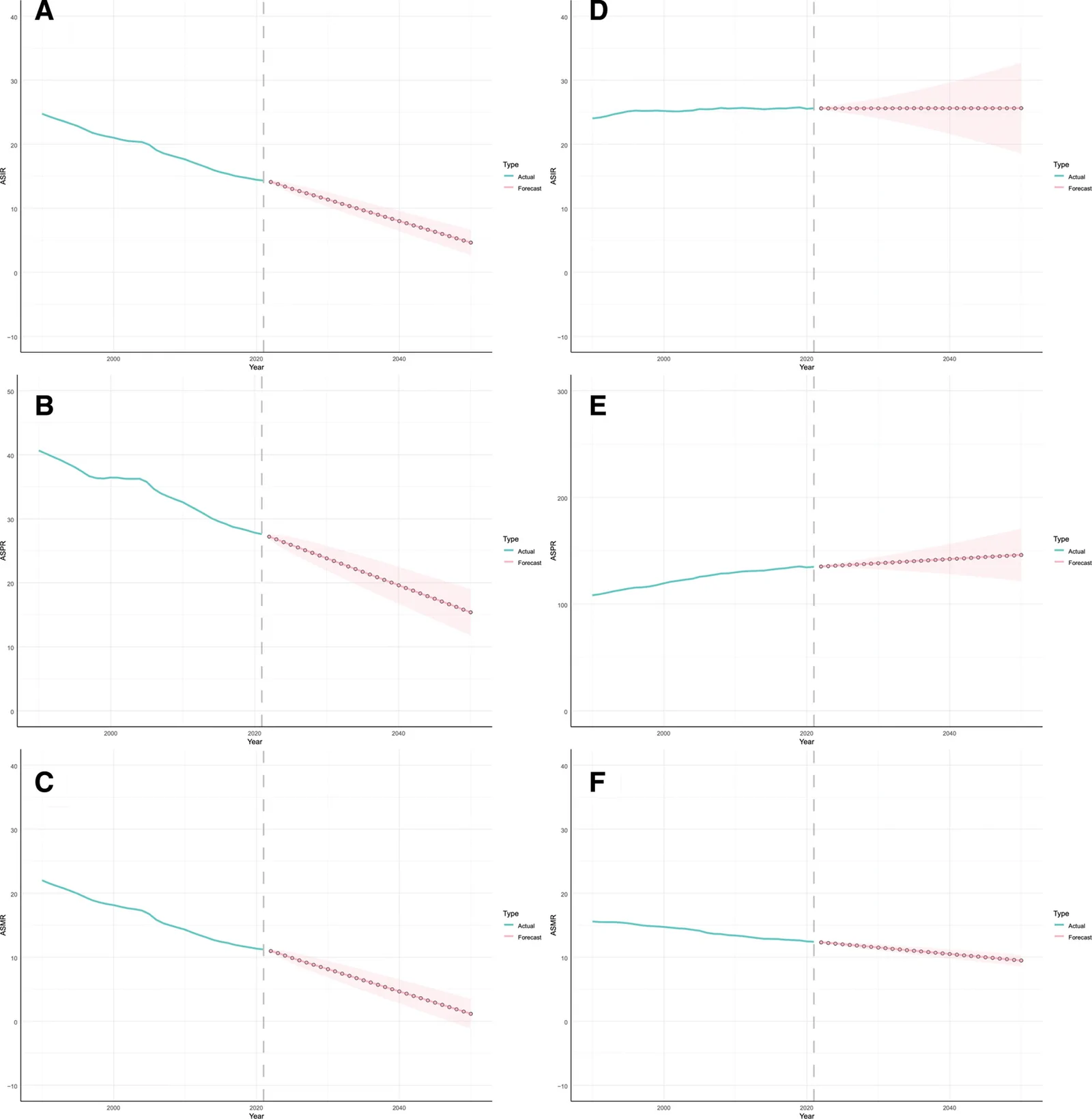
Temporal trends in ASIR, ASPR, and ASMR for gastric cancer (A–C) and colorectal cancer (D–F) from 1990 to 2050. Red dot lines and shaded regions represent the predicted trends and their 95% CI, respectively. ASIR = age-standardized incidence rate, ASMR = age-standardized mortality rate, ASPR = age-standardized prevalence rate, CI = confidence interval.

Given the increasing uncertainty associated with long-term forecasts, projections to 2050 are presented to illustrate overall temporal tendencies rather than to provide precise quantitative estimates. ARIMA-based projections were conducted to explore potential future trends in gastric and colorectal cancer burden from 2021 to 2050 (Fig. 8). Overall, the ASIR and ASMR of gastric cancer are projected to continue declining over the forecast period. For colorectal cancer, ASIR is expected to remain relatively stable or show a modest increase, whereas ASMR is projected to further decrease. Given the inherent uncertainty associated with long-term time-series forecasting, particularly for distant future years, projections are presented in terms of overall trends with corresponding 95% confidence intervals rather than precise point estimates.

## 4. Discussion

Given the model-based and ecological nature of GBD estimates, the findings warrant cautious interpretation. Malignant neoplasms are a leading cause of morbidity and reduced life expectancy worldwide, and digestive system tumors comprise a substantial proportion of the global burden. In 2022, approximately 4.9 million new cases of digestive system cancers were diagnosed globally, accounting for 24.6% of all new cases of cancer. Gastric and colorectal cancers are among the most prevalent digestive tumors and are characterized by high morbidity and mortality rates. In 2022, an estimated 1,926,000 new cases of colorectal cancer were reported globally, positioning it as the third most common cancer of the digestive system. Similarly, in 2022, gastric cancer accounted for approximately 968,350 new cases and 659,850 deaths, ranking fifth globally in terms of incidence and mortality.^[16]^

Gastric and colorectal cancers have emerged as critical global health issues requiring urgent attention and targeted intervention. Using information from the GBD 2021 study, the present study analyzed regional and country-specific patterns between 1990 and 2021, providing up-to-date temporal and geographic insights into the global burden of gastric and colorectal cancers.

ASIR, ASPR, ASMR, and ASDR of gastric cancer showed a sustained declining trend over time, consistent with prior population-based studies and global cancer surveillance reports.^[17,18]^ In 2021, global ASIR and ASMR declined substantially compared with 1990, which may partly reflect improvements in lifestyle and hygiene, including reduced intake of preserved foods and declining prevalence of *H pylori* infection.^[19,20]^ However, despite declining age-standardized rates, the absolute number of gastric cancer cases and deaths continues to increase, which may be associated with population aging, population growth, and lifestyle changes.^[21]^ For colorectal cancer, both ASIR and ASPR demonstrated an increasing trend, whereas ASMR and ASDR showed a decreasing trend. Of note, the increasing trends in ASIR and ASPR of colorectal cancer may be associated with economic and industrial development, the gradual westernization of lifestyles in developing countries, and increased exposure to established risk factors for colorectal cancer in populations born in the second half of the 20th century, such as a high intake of red or processed meat, obesity, physical inactivity, sedentary lifestyle, and early smoking and alcohol use.^[22–28]^

Notably, the global distribution of ASIR in gastric and colorectal cancers was different. The prevalence of gastric cancer is high in East Asia, including Mongolia, Afghanistan, Bolivia, China, and South Korea, whereas that of colorectal cancer is high in several developed countries, including the United States, Canada, and economically developed countries in Europe. In the high-SDI regions, the ASIR, ASMR, and ASDR of gastric cancer all displayed a decreasing trend. In certain low-SDI regions, the declining trend of gastric cancer was less evident, which may be associated with limited healthcare infrastructure and potential under-detection of cases.^[29]^ The higher ASIR of colorectal cancer observed in high-SDI regions may be associated with dietary patterns, including higher consumption of red and processed meat.^[30]^

Globally, Mongolia exhibits one of the highest ASMRs for gastric cancer, which may be associated with the limited availability of endoscopic resources and specialized healthcare services.^[31]^ Early-stage gastric cancer is generally asymptomatic and presents with clinical symptoms of chronic gastritis, posing challenges in diagnosis, resulting in the majority of patients being diagnosed at an advanced stage.^[13]^ Although Japan and South Korea, both high-SDI countries in East Asia, have a high incidence of gastric cancer, their ASMR was not significantly higher compared with other high-SDI countries. This pattern may partly reflect the widespread implementation of gastroscopy-based screening programs in these countries.^[32,33]^

It is worth noting that although the Netherlands had the highest ASIR for colorectal cancer globally, its ASMR and ASDR were not correspondingly elevated. This pattern may be associated with multiple contextual factors, such as the widespread implementation of screening programs, advances in diagnostic and therapeutic modalities, comprehensive staging systems, favorable socioeconomic conditions, high levels of public health awareness, and the overall performance of the healthcare system.^[34–36]^

Importantly, the incidence of gastric cancer was significantly higher in males compared with females. Indeed, the cumulative number of gastric cancer cases in the top 3 high-incidence age groups was 6,603,467 in females and 15,387,470 in males. According to a previous study, estrogen has been suggested to play a protective role against gastric cancer. The “Million Women” research program in the United Kingdom comprised a prospective cohort of 1.31 million women and described that the risk of gastric cancer in postmenopausal women was 1.59 times higher than that in premenopausal women. In addition, among menopausal women, the risk of gastric cancer increased by 1.18-fold for every 5-year decrease in age at menopause.^[37]^

Furthermore, the incidence of colorectal cancer in the top 3 high-incidence age groups totaled 8,480,401 among females and 11,279,253 among males, in line with the finding of a previous study that reported that males are more likely to develop colorectal cancer than females.^[38]^ This may be possibly related to factors such as hormone levels, lifestyle habits, and dietary factors.^[39]^

From a public health perspective, these projected trends may have important implications for cancer prevention strategies and healthcare planning. In the foreseeable future, ARIMA-based projections suggest a potential continued decline in the ASIR of gastric cancer; however, these projections should be interpreted cautiously given the uncertainty inherent in long-term forecasting. Conversely, the ASIR for colorectal cancer is anticipated to remain relatively stable. This pattern may reflect the coexistence of opposing influences, including increased exposure to established risk factors and improvements in medical care and screening practices, although these interpretations should be viewed cautiously, given the ecological and model-based nature of the data.

This study has several limitations that should be acknowledged. First, the ecological design and use of aggregated GBD estimates preclude causal inference at the individual level. In addition, results were interpreted primarily in terms of population-level trends rather than precise estimates for specific years. Second, although GBD modeling incorporates multiple sources to address missing data and improve comparability across settings, residual model uncertainty may persist; therefore, results should be interpreted with appropriate caution, particularly when making comparisons across regions and over time. In addition, the reliability of long-term forecasts may be further affected by unmeasured changes in risk factor distributions, screening practices, and healthcare access over time. Finally, risk factor information was not incorporated into this analysis, and future epidemiological studies using individual-level data are needed to validate and complement these model-based findings.

In future studies, sensitivity analyses could be conducted by triangulating GBD estimates with alternative data sources, such as national cancer registries, vital registration systems, or population-based cohort datasets, and by applying alternative modeling strategies (e.g., Bayesian hierarchical models or ensemble forecasting approaches) to evaluate the robustness of long-term projections.

## 5. Conclusion

Gastric and colorectal cancers are common gastrointestinal tumors that impose a substantial burden on the global healthcare system. However, the incidence of gastric cancer is projected to decline, whereas that of colorectal cancer is expected to remain stable by 2050. Differential prevention, screening, and treatment measures, such as reduced intake of processed meat and endoscopic screening, need to be developed across different regions and populations to prolong the lifespan of the population and concurrently alleviate the public health burden of gastric and colorectal cancers.

## Acknowledgments

The authors express their sincere gratitude to all members who participated in this study.

## Author contributions

**Data curation:** Ling Yu, Wei Xu.

**Formal analysis:** Ling Yu, Wei Xu.

**Software:** Wei Xu.

**Writing – original draft:** Ling Yu.

**Writing – review & editing:** Wei Xu.
